# Identification of an Exopolysaccharide Biosynthesis Gene in *Bradyrhizobium diazoefficiens* USDA110

**DOI:** 10.3390/microorganisms9122490

**Published:** 2021-12-01

**Authors:** Chunxia Xu, Huaqin Ruan, Wenjie Cai, Christian Staehelin, Weijun Dai

**Affiliations:** 1 Guangdong Laboratory for Lingnan Modern Agriculture, Guangzhou 510006, China; 15021922909@163.com (C.X.); caiwenjie@stu.scau.edu.cn (W.C.); 2 Guangdong Province Key Laboratory of Microbial Signals and Disease Control, Integrative Microbiology Research Center, South China Agricultural University, Guangzhou 510642, China; 3 State Key Laboratory of Biocontrol and Guangdong Key Laboratory of Bioresources, School of Life Sciences, Sun Yat-sen University, Guangzhou 510006, China; ruanhuaqin@outlook.com (H.R.); cst@mail.sysu.edu.cn (C.S.)

**Keywords:** exopolysaccharide, *Bradyrhizobium*, plant growth promotion

## Abstract

Exopolysaccharides (EPS) play critical roles in rhizobium-plant interactions. However, the EPS biosynthesis pathway in *Bradyrhizobium diazoefficiens* USDA110 remains elusive. Here we used transposon (Tn) mutagenesis with the aim to identify genetic elements required for EPS biosynthesis in *B. diazoefficiens* USDA110. Phenotypic screening of Tn5 insertion mutants grown on agar plates led to the identification of a mutant with a transposon insertion site in the *blr2358* gene. This gene is predicted to encode a phosphor-glycosyltransferase that transfers a phosphosugar onto a polyprenol phosphate substrate. The disruption of the *blr2358* gene resulted in defective EPS synthesis. Accordingly, the *blr2358* mutant showed a reduced capacity to induce nodules and stimulate the growth of soybean plants. Glycosyltransferase genes related to *blr2358* were found to be well conserved and widely distributed among strains of the *Bradyrhizobium* genus. In conclusion, our study resulted in identification of a gene involved in EPS biosynthesis and highlights the importance of EPS in the symbiotic interaction between USDA110 and soybeans.

## 1. Introduction

Establishment of an effective nodule symbiosis between nitrogen-fixing rhizobia and host plants depends on reciprocal complex molecular dialogues. In many interactions, nodule formation requires rhizobial exopolysaccharides (EPS) or EPS-derived oligosaccharides [1,2,3,4,5,6,7]. For example, the EPS of *Bradyrhizobium diazoefficiens* (*B. japonicum*) USDA110 was found to play an important role in the symbiosis with soybean plants [8,9,10]. However, EPS is dispensable for the symbiosis between *Sinorhizobium* (*Ensifer*) *fredii* HH103 and soybeans [11]. In the interaction between *Mesorhizobium loti* and *Lotus japonicus,* EPS is conditionally required during different symbiotic stages [12]. Likewise, EPS oligosaccharides produced by *Sinorhizobium* sp. NGR234 are essential for the nodulation of various legumes but dispensable for the nodulation of cowpea [13]. The absence of EPS or the synthesis of altered oligosaccharides can impair the rhizobial root hair infection process, suggesting that EPS contributes to the suppression of plant defense reactions in compatible interactions [14]. EPS can protect rhizobia and other bacteria from biotic and abiotic stresses by influencing the formation of bacterial biofilms, adhesion to root surfaces, and other mechanisms [2,3]. Moreover, EPS or oligosaccharides derived from EPS possess a signaling role during rhizobial infection. The LysM receptor kinase EPR3 in *L. japonicus* has been identified as a first EPS receptor, suggesting that EPS affects symbiotic signaling in host plants [12].

Bacterial EPS synthesis is initiated by a priming glycosyltransferase that transfers a phosphosugar onto a polyprenol phosphate substrate. The galactosyltransferase ExoY of *S. meliloti* and the glucosyltransferase PssA of *Rhizobium leguminosarum* are examples of such phosphor-glycosyltransferases [15,16]. The membrane-bound product serves as a substrate for additional glycosyltransferases which catalyze the stepwise addition of monomeric carbohydrate units. The formed EPS repeating subunit is then flipped across the inner bacterial membrane. Finally, the EPS repeating subunit is polymerized and exported [4,15]. EPS production is ubiquitous in rhizobia, which typically synthesize EPS repeating units consisting of branched oligosaccharides. The type of sugars, their linkage in the single subunit, the size of the subunit, the polymerization degree, and non-carbohydrate decorations differ in the EPS of rhizobia [2,3,17]. The EPS of *B. diazoefficiens* USDA110 is composed of mannose, 4-*O*-methylgalactose (or galactose), glucose, and galacturonic acid [18,19]. The EPS structures of *B. japonicum* USDA123 produced in cultures and by bacteroids in soybean nodules are different. The nodule polysaccharide contains galactose, rhamnose, and 2-*O*-methylglucuronic acid. In contrast, the EPS from *B. elkanii* cultures and bacteroids is identical and composed of rhamnose and 4-*O*-methylglucuronic acid [20].

Rhizobial genes required for synthesis of EPS (*exo* genes) have been identified in various rhizobia including *Sinorhizobium* (*Ensifer*) *meliloti*, *Rhizobium leguminosarum* bv. *trifolii*, *Mesorhizobium loti*, and *Sinorhizobium* strains [1,4,13,21]. EPS gene expression is regulated in a complex way and varies in different rhizobia [4,21]. A few genes involved in the EPS biosynthesis pathway have been also identified in *B. diazoefficiens* USDA110, namely an *exo* gene cluster containing *exoU*, *exoM metA*, *exoP (blr7576)*, *exoT*, and *exoB* genes [10,21] and a second gene cluster containing *lspL-ugdH* [9] and putative additional *exo* genes [22]. The expression of *exo* genes in the second gene cluster was upregulated in response to soybean seed extracts [23].

Mutants with deletions in the first *exo* gene cluster inoculated on soybeans showed a considerably reduced nodulation and displayed reduced nodulation competitiveness [8,10]. An *exoB* insertion mutant of USDA110 (lacking UDP galactose synthesis) produced EPS without galactose and only empty pseudonodules were observed on inoculated soybean roots [9]. Similarly, a *lspL-ugdH* deletion mutant of USDA110 produced EPS without galacturonic acid and showed reduced competitiveness in nodulation experiments with soybeans [9]. However, most bradyrhizobial EPS synthesis genes have not yet been discovered.

In this study, we performed a genetic screen with a Tn5-derived library of *B. diazoefficiens* USDA110, aiming to identify transposon (Tn5) insertion mutants that are defective in EPS production. The screen resulted in identification of *blr2358*, a putative phosphor-glycosyltransferase gene in the second *exo* gene cluster containing *lspL*-*ugdH*. The inactivation of *blr2358* by the Tn5 insertion was associated with defective EPS production and altered colony morphology. When inoculated on soybeans, reduced plant growth-promoting effects were observed. A sequence analysis revealed that *blr2358*-related glycosyltransferases are conserved in the *Bradyrhizobium* genus and that predicted homologs are redundant in *Bradyrhizobium* genomes. Our study suggests that Blr2358 participates in EPS biosynthesis, and therefore affects the symbiotic interaction between *B. diazoefficiens* and soybeans.

## 2. Materials and Methods

### 2.1. Primers, Plasmids, and Bacterial Strains

*Bradyrhizobium diazoefficiens* USDA110 was a present from Hauke Hennecke [24]. The USDA110 pLG107-Tn5-based mutant library was obtained from Caroline Harwood (University of Washington, Seattle, WA, USA). If not otherwise specified, the USDA110 parent strain and the mutant derivatives were grown in arabinose–gluconate (AG) medium containing 1.0 g yeast extract, 1.0 g L-arabinose, and 1.0 g Na-gluconate per liter (pH 7.0) [25] at 28 °C. Primers used in this study are shown in Supplementary Table S4. Strains and plasmids are listed in Supplementary Table S5.

### 2.2. Tn5 Library Screening

The Tn5 mutant library stored at −80 °C was recovered on AG agar plates at 30 °C. The parent strain USDA110 and the Tn5 mutant colony were detected on AG agar plates containing 60 mg/L of Congo red at 30 °C. The bacterial morphology was photographed 5 days post inoculation.

### 2.3. Whole-Genome Short Read Re-Sequencing

One μg of microbial genomic DNA was sonicated to an average size of ~350 bp by a Covaris-S220 ultrasonicator (Covaris, Woburn, MA, USA). Illumina DNA fragment library preparation was performed following the manual of the Next-Generation Sequencing DNA library preparation kit (Novagen, Tianjin, China). Briefly, the sonicated DNA products were end-repaired and ligated with an adapter. Adapter-ligated products were purified using AMPure XP beads (Agencourt-Berkman Coulter, IN, USA) and enriched through PCR amplification using the custom adapter-specific primers. The obtained unbiased short read library was further cleaned up with AMPure XP beads. Pair end Illumina HiSeq PE150 sequencing was performed with an Illumina NovaSeq 6000 sequencing system.

### 2.4. Identification of Tn5 Insertion Sites

Raw short reads were subjected to quality control including removing adapters using cutadapt (v1.16) by Novagen, yielding clean short reads. Clean short reads were mapped to the *B. diazoefficiens* USDA110 reference genome (NC_004463.1) with the BWA (v0.7.15-r1140) software. Unmapped reads containing a Tn5 sequence were extracted and assembled into longer contigs using Samtools (v1.5), KmerGenie (v1.7044), and Minia (v2.0.7). These assembled contigs were further subjected to BLAST analysis against the reference genome of *B. diazoefficiens* USDA110 (NC_004463.1) and the Tn5 sequence (pLG107, KF532966.1), using BLAST+ software (https://blast.ncbi.nlm.nih.gov/Blast.cgi?CMD=Web&PAGE_TYPE=BlastHome, assessed 2 March 2018). The right contigs containing genome-Tn5-genome sequences had double BLAST hits.

### 2.5. Used Software

The following software was used for sequence analysis.

Miniconda software (https://conda.io/miniconda.html, assessed 1 March 2018); BWA software, version 0.7.15-r1140 (http://bio-bwa.sourceforge.net, assessed 1 March 2018) [26]; cutadapt software, version 1.16 (https://github.com/marcelm/cutadapt/, assessed 1 March 2018) [27]; Samtools software, version 1.5 (http://samtools.sourceforge.net, assessed 1 March 2018) [28]; BLAST+ software, version 2.7.1 (https://blast.ncbi.nlm.nih.gov/Blast.cgi?CMD=Web&PAGE_TYPE=BlastHome, assessed 1 March 2018) [29]; Minia software, version v2.0.7 (http://minia.genouest.org/, assessed 1 March 2018) [30]; and KmerGenie software, version 1.7044 (http://kmergenie.bx.psu.edu/, assessed 1 March 2018) [31]. Operon prediction was performed with MicrobesOnline software (http://www.microbesonline.org/operons/, assessed 15 November 2021) and the corresponding promoter prediction with BDGP software (https://www.fruitfly.org/seq_tools/promoter.html, assessed 15 November 2021).

### 2.6. Nodulation Tests with Soybean Plants

A Leonard jar assembly [32] was used to assess nodule formation and plant growth-promoting effects caused by USDA110 and mutant bacteria. The upper jar was filled with sterilized soil (1/3) and sterilized vermiculite (2/3), while the lower jar contained nitrogen-free nutrient solution (Fåhraeus medium) [33]. The whole jar units were autoclaved before use. Soybean seeds were surface sterilized with 75% ethanol for 10 min and then washed with sterilized H_2_O three times. Seeds were further treated with a 5% sodium hypochlorite solution for 5 min and washed with H_2_O at least ten times. Sterilized seeds were soaked with H_2_O for 24 h and subsequently germinated on 0.2% (*w*/*v*) agar plates at 22 °C. Germinated seeds were then transferred into the upper jars (1 plant per jar). For inoculation with bradyrhizobia, 1 mL of a bacterial suspension suspended in 10 mM of MgSO_4_ (bacterial growth reaching late logarithmic phase; OD_600_ ≈ 1.5) was added to each jar. Plants were cultivated at 24 ± 2 °C under a 16 h light/8 h dark cycle at a light intensity of 18,000 lux. More than three experiments (biologically independent repeats) were performed, and each experiment (6 plants per strain) contained at least six technical repeats.

### 2.7. Complementation of the Blr2358 Mutant

The *blr2358* gene was cloned into the pBBR1MCS-5 vector (gentamycin resistance) [34] using the Vazyme ClonExpress II One Step Cloning kit (Vazyme Biotech, Nanjing, China), generating theplasmid pBBR1-MCS-*blr2358*. The plasmid pBBR1-MCS-*blr2358* was mobilized into the *blr2358*::Tn mutant through electroporation, resulting in the complementation strain *blr2358*::Tn + *blr2358*. The primers used for cloning are listed in Supplementary Table S4.

### 2.8. Ethanol Precipitation of Culture Supernatants and Anthrone Tests

*B. diazoefficiens* USDA110 and the *blr2358* mutant strain were cultured in liquid AG medium for 5 days at 28 °C. Bacteria (OD_600_ ≈ 1.00) were then transferred to defined GMS medium (5 g/L mannitol, 1 g/L glutamic acid, 6 mM K_2_HPO_4_; adjusted with KOH to pH 7 and then supplemented with 1 mM MgSO_4_, 250 μM CaCl_2_, 100 g/L MnCl_2_·4H_2_O, 10 g/L H_3_BO_3_, 10 g/L ZnSO_4_·7H_2_O, 10 g/L CoCl_2_·6H_2_O, 10 μg/L CuSO_4_·5H_2_O, 2.5 mg/L FeCl_3_·6H_2_O, and 1 mL/L Gamborg’s B5 vitamin solution) (Sigma-Aldrich, St. Louis, MO, USA). Harvested cultures were adjusted to OD_600_ ≈ 1.0 and centrifuged. The supernatant of each 100 mL cell culture was mixed with 500 mL of 100% ethanol. After overnight incubation at 4 °C, precipitates were collected by centrifugation. The pellets were then air dried and washed 3 times with 83% ethanol. Finally, the material was dissolved in 10 mL of H_2_O and quantified by the anthrone method.

### 2.9. Determination of Carbohydrate Contents

Carbohydrate contents of ethanol precipitates were quantified with the anthrone method as described previously [35]. Samples were dissolved in H_2_O and aliquots (30 μL) were mixed with 83 μL of anthrone reagent (0.01 g anthrone, 0.5 mL ethyl acetate, and 5 mL concentrated H_2_SO_4_). Samples were incubated in a water bath at 100 °C for 4 min and then cooled on ice for 10 min. The optical density of the samples was measured at 620 nm with a microplate reader (Synergy H1MF, BioTek Instruments, Winooski, VT, USA). Known glucose concentrations served as a reference.

### 2.10. Preparation of Oligosaccharides from Ethanol Precipitated Polysaccharide

Ethanol precipitated material was air-dried and then dissolved in a volatile buffer (100 mM pyridine/acetic acid buffer, 8.07 mL pyridine, 5.724 mL acetate, and 986.206 mL H_2_O; pH 5.0). Subsequently, the sample was passed through a Detoxi-Gel^TM^ Endotoxin Removing Gel column (Thermofisher, IL, USA) to remove lipophilic compounds. The sample was then loaded on a Sephadex G-75 gel filtration column (GE Healthcare Life Sciences, Marlborough, UK) equilibrated with the same volatile buffer. The high-molecular faction was collected and lyophilized to remove the volatile pyridine/acetic buffer. The sample (≈50 μg) was then dissolved in 200 μL of H_2_O and mixed with 200 μL of 4 M trifluoroacetic acid (TFA). After incubation in a boiling water bath for 3.5 h, the hydrolyzed mixture was cooled down on ice and the pH was adjusted to 5 with NaOH. The aliquot was then passed through a Dowex 50 W × 4–100 column (H^+^ form; treated with 2 M HCl for 3 h and equilibrated with H_2_O). Finally, the oligosaccharides were eluted with H_2_O and purified on a Sephacryl S-100 gel filtration column (GE Healthcare Life Sciences, Marlborough, UK) by using an AKTA protein purification system (GE Healthcare Life Sciences, Marlborough, UK). Pyridine/acetic acid buffer served as the running solvent. The low-molecular fraction containing oligosaccharides was identified with the anthrone method, lyophilized, and subjected to mass spectrometry analysis.

### 2.11. Mass Spectrometry Analysis of Oligosaccharides

Matrix-assisted laser desorption/ionization time-of-flight mass spectrometry (MALDI-TOF MS) was used to analyze the oligosaccharides obtained by TFA hydrolysis. To prepare the matrix, 1 mg/mL of 2,3-dihydroxybenzoic acid (Sigma-Aldrich, St. Louis, MO, USA) was added to 50% acetonitrile supplemented with 0.1% (*w*/*v*) trifluoroacetic acid. The oligosaccharide sample (≥20 μg) was dissolved in H_2_O and mixed with the same volume of matrix. The test mixture (2 μL) was applied to an MTP 384 ground steel plate and air-dried. MALDI-TOF MS analysis was performed with an Ultraflex III MALDI-TOF mass spectrometer (Bruker Daltonics, Billerica, MA, USA) in the positive ionization mode. The accelerating voltage of the ion source was 2.5 kV and the accelerating voltage of the reflector was 26.3 kV. The sample was ionized with a Smartbeam 1 ultraviolet laser (λ = 355 nm) [36].

### 2.12. Analysis of Blr2358 Homologs

The amino acid sequences of Blr2358 (NP_768998) were used to blast the genomes of the *Bradyrhizobium* genus in the IMG database (https://img.jgi.doe.gov/, assessed 5 October 2021) using default parameters (BLASTP, E-value: 1 × 10^−5^). A protein domain search was performed for the sugar transferase domain (pfam02397) in the IMG database.

### 2.13. Phylogenetic Tree Analysis

The 16S ribosomal RNA gene sequences of representative strains of the *Bradyrhizobium* genus were extracted from the IMG database, assessed 7 October 2021. A phylogenetic tree of 16S rDNA sequences was constructed by MEGA6 using the neighbor-joining method with 1000 bootstrap replications.

### 2.14. Statistical Analysis

Statistical analyses were performed using Excel and R software (http://www.R-project.org/, assessed 2 February 2021).

## 3. Results

### 3.1. Phenotypic Screening of a B. diazoefficiens USDA110 Transposon Mutant Library

To identify genetic elements of the entire genome involved in polysaccharide biosynthesis, we conducted a Tn5 mutagenesis screen in *B. diazoefficiens* strain USDA110. We took advantage of a previously created Tn5-based random insertion USDA110 mutant library [37]. We first conducted a preliminary search for suitable media and found that AG medium supplemented with Congo red was the most suitable for displaying EPS production. In our screening assay, wild-type USDA110 colonies exhibiting a mucoid morphology served as a control. Tn5-inserted colonies showing a non-mucoid phenotype and a reduced colony size were considered as potential EPS mutants. A total of 50,000 Tn5 mutant colonies were screened, and 20 colony candidates with a dry, non-mucoid phenotype were isolated. Three colonies with the strongest non-mucoid phenotype were chosen for further investigation.

### 3.2. Identification of Tn5 Insertion Sites

We next sought to identify the precise Tn5 insertion sites in the obtained mutant strain candidates. Instead of the conventional HiTAIL-PCR method [38], our study used a whole genome short-read re-sequencing (WGS) method to determine Tn5 localizations. Single or multiple Tn5 insertion sites in those mutant colonies were identified by a customized bioinformatics pipeline and Tn5 flanking sequences were then confirmed by Sanger sequencing. Three mutants (BJ#25, BJ#47, and BJ#57) were found to bear a Tn5 insertion in gene *blr2358*, and both BJ#47 and BJ#57 contained another Tn5 insertion in a *leuC* gene (*bl10416*) (Table 1 and Table S1). As a Tn5 insertion in gene *blr2358* was common in these three mutants, we inferred that gene *blr2358* is most likely responsible for the non-mucoid phenotypes observed in the mutant colonies. The *blr2358* gene is predicted to encode a phosphor-glycosyltransferase family protein (WP_011085145.1). The following experiments were therefore focused on the *blr2358* gene (colony BJ#25).

### 3.3. Disruption of the Blr2358 Gene Impairs EPS Production

**Figure 1:**
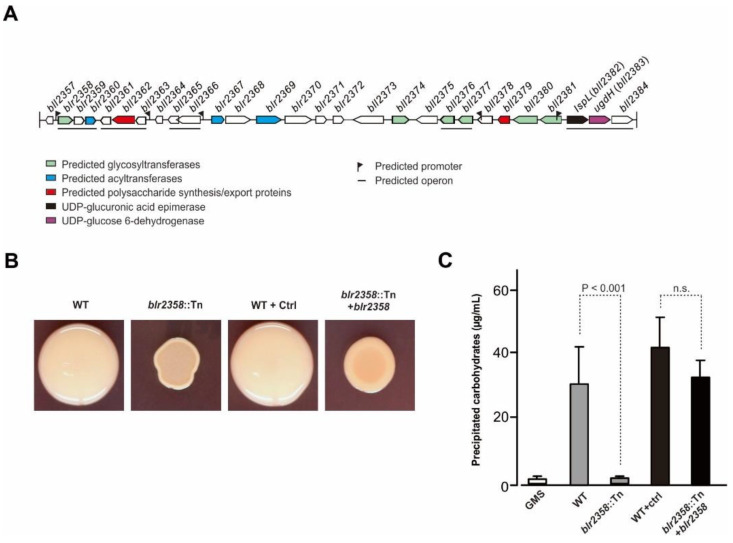
The *blr2358* gene of *B. diazoefficiens* USDA110 is related to polysaccharide biosynthesis. (**A**) Gene cluster of *B. diazoefficiens* USDA110 containing the *blr2358* gene. (**B**) Morphology of Tn5-inserted *blr2358* mutant colonies (*blr2358*::Tn) as compared to the wild-type strain USDA110 (WT). USDA110 carrying the empty pBBR1-MCS5 plasmid (WT + Ctrl) and the mutant carrying pBBR1-MCS5-*blr2358* (*blr2358*::Tn + *blr2358*) are also shown. Equal amounts of bacteria were spotted on an AG plate containing Congo red and colonies were photographed after incubation at 30 °C for 5 days. (**C**) Quantification of carbohydrates in ethanol precipitates obtained from supernatants of cell cultures. WT, *blr2358*::Tn mutant, WT carrying an empty control plasmid, and *blr2358*::Tn mutant complemented with a *blr2358* copy were assayed. Ethanol precipitates from GMS medium without bacteria were also analyzed. Data (means ± SD; *n* = 6; *t*-test) were expressed as μg precipitated carbohydrates (glucose equivalents) per mL bacterial culture supernatant (from bacterial suspensions adjusted to OD_600_ ≈ 1.00).

The *blr2358* gene is located in a large gene cluster with coordinates 2,561,178 to 2,595,273 (*blr2358*–*bll2384*; [9,22] (Figure 1A). The gene cluster contains predicted polysaccharide synthesis genes, namely *blr2358* and five other glycosyltransferase genes (*blr2374*, *bll2376*, *bll2377*, *bll2380*, and *bll2381*), three acyltransferase genes (*blr2360*, *blr2367*, and *blr2369* named *exoZ* in Kaneko et al. 2002), and two genes predicted to be involved in the polymerization and export of a polysaccharide, namely *blr2362* (named *exoP* in Kaneko et al. 2002) and *bll2379*. In addition, the cluster contains *lspL* (*blr2382*; UDP-glucuronic acid epimerase gene) and *ugdH* (*blr2383*; UDP-glucose 6-dehydrogenase gene), which, when deleted, lead to the formation of EPS lacking galacturonic acid [9]. Bioinformatic analysis suggested that *blr2358*,* blr2359* and *blr2360* form an operon and that the expression of these genes is controlled by a promoter upstream of *blr2358.*

The colony morphology phenotype of the BJ#25 mutant supported the view that the *blr2358* gene is implicated in EPS synthesis. Compared to the mucoid colony phenotype of the parent strain, the non-mucoid colonies of #BJ25 containing Tn5-inserted *blr2358* were relatively small in size and dry on AG agar plates (Figure 1B). Furthermore, the complementation of the Tn5-inserted *blr2358* mutant by overexpression of an episomal copy of *blr2358* restored polysaccharide production to a certain extent (Figure 1B). When culture supernatants of the wild-type strain and the complementation strain (grown in defined GMS liquid medium) were precipitated with ethanol, a clear precipitate was formed, while nearly no material was precipitated from culture supernatants of the mutant (Supplementary Figure S1 in Supplementary Information). Anthrone test results indicated that the ethanol precipitate from the wild-type and the complementation strain contained considerable amounts of carbohydrates. In contrast, much less carbohydrates were quantified for the corresponding samples of the mutant strain, reaching a level close to control samples, i.e., GMS medium (without bacteria) treated with ethanol (Figure 1C). These findings provided indications that the gene product of *blr2358* is involved in the synthesis of an extracellular polysaccharide.

To confirm that the carbohydrate-rich ethanol precipitate from the wild-type culture supernatant is indeed EPS (Supplementary Information, Figure S2A), we performed a mass spectrometry (MALDI-TOF MS) analysis for oligosaccharides obtained by the hydrolysis of EPS with TFA. We found that the obtained mass spectrometry signals largely correspond to the known EPS structure of strain USDA110 (Supplementary Information, Figure S2B and Table S2). These findings support the view that the Tn5-inserted *blr2358* mutant is impaired in EPS synthesis.

### 3.4. Soybeans Inoculated with the Blr2358 Mutant Show Reduced Nodule Formation

**Figure 2:**
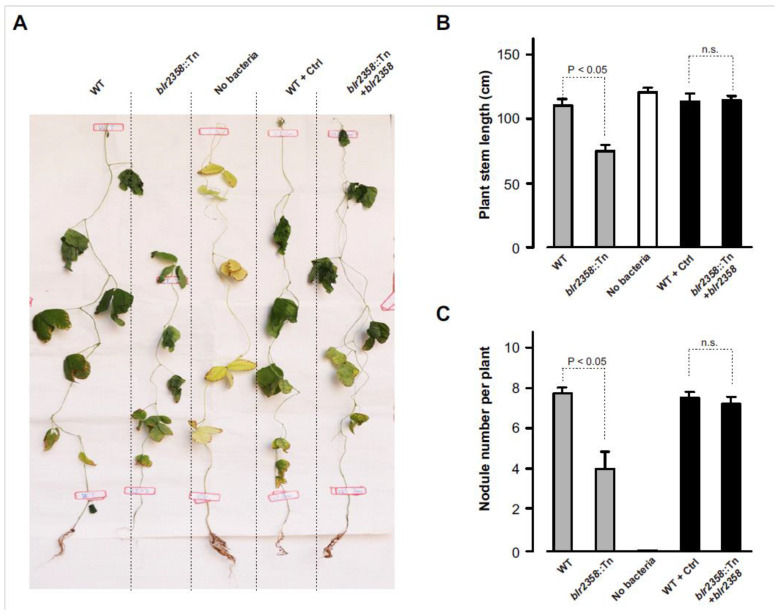
The *blr2358* mutant shows reduced nodule formation. (**A**) Representative pictures of soybean cv. Huaxia No. 3 inoculated with *B. diazoefficiens* USDA110 and indicated mutant strains. Plants were photographed 8 weeks post inoculation. (**B**) Stem length of plants at the time of harvest. (**C**) Number of formed nodules per plant at the time of harvest. Experiments were repeated twice and yielded similar results. Data represent means ± SD (*n* = 6, *t*-test). Abbreviations: WT, wild-type *B. diazoefficiens* USDA110; *blr2358*::Tn, mutant with Tn5 insertion in *blr2358*; No bacteria, no bacteria were used for inoculation; WT + Ctrl, wild-type *B. diazoefficiens* USDA110 carrying the empty pBBR1-MCS5 plasmid; *blr2358*::Tn + *blr2358*, the *blr2358*::Tn mutant carrying pBBR1-MCS5-*blr2358*.

Nodulation tests with soybean cv. Huaxia No. 3 were performed to investigate the symbiotic role of the *blr2358* gene. Seedlings were inoculated with the USDA110 wild-type strain, the *blr2358*::Tn mutant, and the complementation strain (*blr2358*::Tn mutant with a functional copy of *blr2358*). The wild-type strain carrying the corresponding empty plasmid without *blr2358* and mock-inoculated plants were included into the nodulation tests. At 8 weeks post inoculation, plants were harvested and phenotypically analyzed. As expected, the wild-type strain strongly promoted plant growth. Nodulated plants showed green leaves while leaves of mock-inoculated control plants were yellow. In comparison to the wild-type strain, inoculation with the *blr2358*::Tn mutant caused a significantly lower plant stem length and the number of formed nodules was reduced to half. In contrast, the complementation strain showed a symbiotic phenotype that was similar to the wild-type (Figure 2). These findings indicate that *blr2358* disruption in the USDA110 genome negatively affected nodulation and plant growth in the symbiotic interaction between USDA110 and soybeans.

### 3.5. Glycosyltransferase Genes Homologous to Blr2358 Are Conserved in the Bradyrhizobium Genus

**Figure 3:**
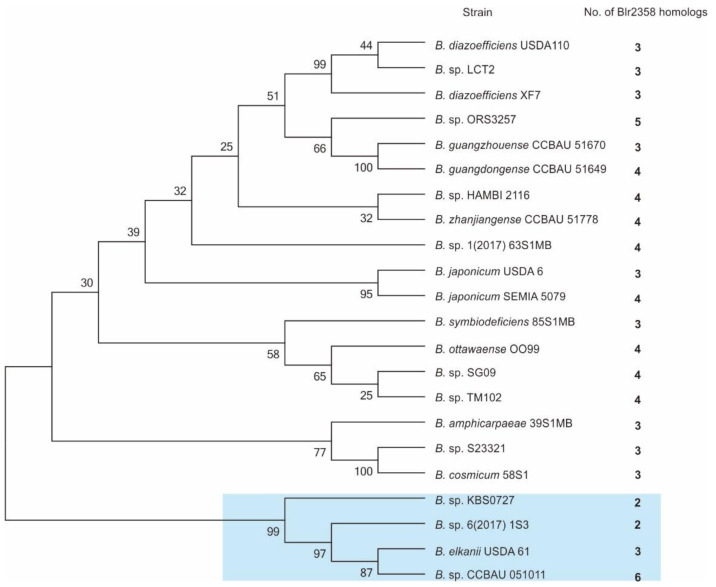
Representative Blr2358 homologs in the *Bradyrhizobium* genus. A phylogenetic tree was made with MEGA6 software using the neighbor-joining method and 16S rDNA sequences from representative *Bradyrhizobium* strains. The number of Blr2358 homologs in each strain is shown. The blue rectangle indicates the clade of *Bradyrhizobium* strains that possess relatively low amino acid sequence similarities to Blr2358.

The number of homologous genes in a bacterial genome can give indications of their functions. We searched for *blr2358* gene homologs in the whole genome of *B. diazoefficiens* USDA110 and found two other glycosyltransferases, which share 31% and 36% amino acid sequence identity with Blr2358. Both proteins contain a sugar transferase domain (pfam02397) (Table S3) and may both function as phospho-glycosyltransferases. The search for Blr2358 homologs was further extended to the *Bradyrhizobium* genus. The results revealed that homologs are widely distributed within the 22 surveyed *Bradyrhizobium* strains (Figure 3). Amino acid sequence similarity ranged from 29% to 96%, with a minimum of two homologs found in *Bradyrhizobium* sp. KBS0725 and *Bradyrhizobium* sp. KBS0725, while a maximum of six homologs were identified in *Bradyrhizobium* sp. CCBAU 051011. Interestingly, a 16S rDNA-based phylogenetic tree analysis revealed that a small phylogroup containing four *Bradyrhizobium* strains is characterized by homologs with a relatively low similarity to Blr2358, i.e., ranging from only 31% to 50% (Figure 3 and Table S3). These findings suggest that phospho-glycosyltransferases are associated with adaptive evolutionary scenarios in this clade. In general, the Blr2358 homologs were found to be conserved and broadly distributed across the *Bradyrhizobium* genus, suggesting that some of them may play a role in symbiotic adaptation to host plants.

## 4. Discussion

We found in this study that a Tn5 insertion in the *blr2358* gene of *B. diazoefficiens* USDA110 correlates with a non-mucoid colony phenotype, suggesting that Blr2358 is involved in polysaccharide synthesis. Blr2358 and homologs are expected to be phospho-glycosyltransferases that transfer a phosphosugar onto a polyprenol phosphate substrate, i.e., Blr2358 is predicted to be responsible for the first step of EPS synthesis. However, based on the EPS structure of *B. diazoefficiens* USDA110 [18,19] (Figure S2A), there are neither glucose nor galactose residues at the reducing end of the EPS subunit and future biochemical research is required to identify the phosphosugar preference of Blr2358. It is worth noting in this context that EPS synthesis was not fully restored by the complementation strain in the performed agar plate assay. This could be explained by the reduced growth of the complementation strain under the used test conditions (plasmid copy number effects) or the production of an altered EPS. Indeed, it is possible that the Tn5 insertion in *blr2358* has certain polar effects on downstream genes, namely *blr2359* and *blr2360.* The *blr2359* gene encodes a putative glycosyl hydrolase which could eventually play a role in processing synthesized EPS subunits prior to polymerization. The *blr2360* gene is predicted to encode an acyltransferase which could be involved in *O*-acetylation of the EPS subunit. These three genes are organized in the same transcriptional direction and may form an operon controlled by a promoter upstream of *blr2358* (Figure 1A). Future research will be required to characterize a complementation strain expressing all three genes.

Structurally different EPS produced by different rhizobial strains may affect the nodulation of soybeans differently. In the case of *S. fredii* HH103, for example, an *exoA* mutant deficient in EPS synthesis induced effective nodules on soybean roots similar to the wild-type strain and even showed an increased competitive capacity to occupy nodules [11]. In contrast, EPS mutants of *B. diazoefficiens* USDA110 were found to be impaired in the symbiosis with soybeans [8,9,10]. In our study, soybean plants inoculated with the *blr2358* mutant showed a significant decrease in nodule formation. Our findings support the view that EPS plays a positive role in the symbiotic relationship between USDA110 and soybeans. Our results also agree with a previous study that suggested a positive role of EPS produced by the *B. japonicum* strain USDA138 [39]. The distinct roles of different EPS could be explained by the discovery that host plants can sense EPS oligosaccharides produced by different rhizobial strains. The EPR3 receptor of *L. japonicus* can recognize EPS oligosaccharides of *M. loti* and that of other strains and hence activate host processes that control rhizobial root-hair infection [40,41,42].

Occurrence of genes closely related to *blr2358* is rather common in bacterial genomes, including strain USDA110. It is expected that these genes encode phospho-glycosyltransferases. The number and variations of these genes in a genome may reflect the diversity of produced polysaccharides. In our study, searching for *blr2358*-encoding homologs in the USDA110 genome yielded two homologs (Table S3 and Figure 3). A certain redundancy of the EPS biosynthesis pathway has been observed for the *exoB* gene, which codes for UDP-glucose-4-epimerase and shares sequence similarity with the *galE* gene of *B. japonicum* 61A101C [43]. Despite possible functional redundancy of homologs, both an *exoB* mutant [8] and the *blr2358* mutant of this study exhibited a clear phenotype, suggesting that homologs may be implicated in processes other than EPS synthesis. Further research on different *Bradyrhizobium* strains will be required to investigate the roles of Blr2358 and homologs in polysaccharide synthesis and other processes under free living conditions and in symbiosis with soybean.

## Figures and Tables

**Table 1:** Identification of transposon insertion sites in *B. diazoefficiens* USDA110 mutant colonies.

Mutant Colony	Insertion Position	Targeted Genes	Annotated Functions
BJ#25	2561969	*blr2358*	Glycosyltransferase family protein
BJ#47	449780	*bll0416 (leuC)*	3-isopropylmalate dehydratase large subunit
	2561969	*blr2358*	Glycosyltransferase family protein
BJ#57	449973	*bll0416 (leuC)*	3-isopropylmalate dehydratase large subunit
	2561772	*blr2358*	Glycosyltransferase family protein
Ctrl	-	-	-

Ctrl, wild-type *Bradyrhizobium diazoefficiens* USDA110 without Tn5 transposon insertions.

## Data Availability

Short read sequencing data sets have been deposited in the Sequence Read Archive (SRA) (www.ncbi.nlm.nih.gov/sra, assessed 5 March 2018) under accession number SRP148518.

## Supplementary Materials

The following are available online at https://www.mdpi.com/article/10.3390/microorganisms9122490/s1, Figure S1. Photograph of ethanol precipitates obtained from culture supernatants of B. diazoefficiens USDA110, the blr2358 mutant and a complementation strain; Figure S2: MALDI-TOF spectrum of oligosaccharides of B. diazoefficiens USDA110 derived from the ethanol-precipitated polysaccharide; Table S1; Statistics of whole-genome short read re-sequencing results obtained from Tn5 mutant colonies. Table S2: Predicted and observed mass spectrometry signals obtained from the MALDI-TOF spectrum shown in Figure S2. Table S3: List of glycosyltransferase homologs of blr2358 in the Bradyrhizobium genus; Table S4: Oligonucleotides used in this study. Table S5: Bacterial strains and plasmids used in this study.
