# Supplementary material for: Identification of an Exopolysaccharide Biosynthesis Gene in Bradyrhizobium diazoefficiens USDA110
Source: Microorganisms. 2021 Dec 1;9(12):2490. doi: 10.3390/microorganisms9122490 (PMC8707904; doi:10.3390/microorganisms9122490)
Supplement: Supplementary file 1 [file microorganisms-09-02490-s001.zip › Supplementary Information_2nd Revision CS241121.pdf]

Supplementary Information

Identification of an exopolysaccharide biosynthesis gene in *Bradyrhizobium diazoefficiens* USDA110

Chunxia Xu, Huaqin Ruan, Wenjie Cai, Christian Staehelin and Weijun Dai

Figure S1. Photograph of ethanol precipitates obtained from culture supernatants of *B. diazoefficiens* USDA110 the *blr2358* mutant and a complementation strain.

Figure S2. MALDI-TOF spectrum of oligosaccharides of *B. diazoefficiens* USDA110 derived from the ethanol-precipitated polysaccharide.

Table S1. Statistics of whole-genome short read re-sequencing results obtained from Tn5 mutant colonies.

Table S2. Predicted and observed mass spectrometry signals obtained from the MALDI-TOF spectrum shown in Figure S2.

Table S3. List of glycosyltransferase homologs of *blr2358* in the *Bradyrhizobium* genus. See separate Excel file.

Table S4. Oligonucleotides used in this study.

Table S5. Bacterial strains and plasmids used in this study.

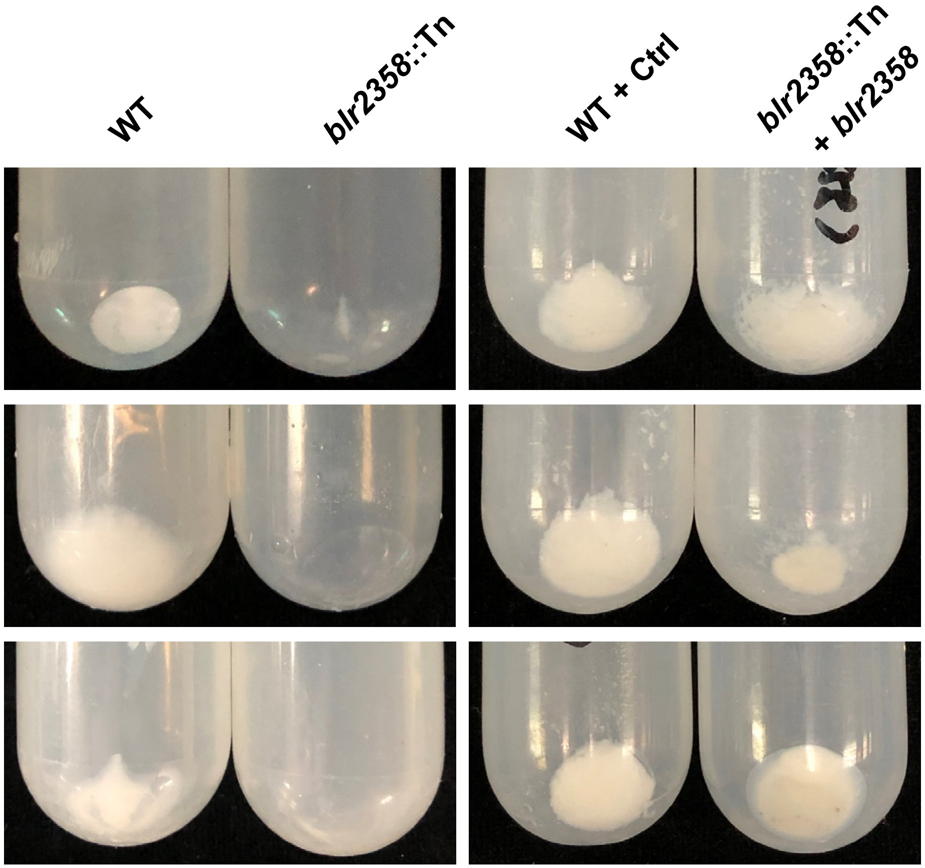

Figure S1. Photograph of ethanol precipitates obtained from culture supernatants of *B. diazoefficiens* USDA110, the *blr2358* mutant and a complementation strain. Three biological replicates of each strain were grown in GMS medium and culture supernatants (cell cultures adjusted to OD_{600}≈1.0) were treated with ethanol at a ratio of 1:5. After overnight incubation at 4°C, precipitated material was obtained by centrifugation. Abbreviations: WT, wild-type *B. diazoefficiens* USDA110; *blr2358*::Tn, mutant with Tn5 insertion in *blr2358*. WT + Ctrl, WT carrying an empty control plasmid; *blr2358*::Tn + *blr2358,* *blr2358*::Tn mutant complemented with a *blr2358* copy.

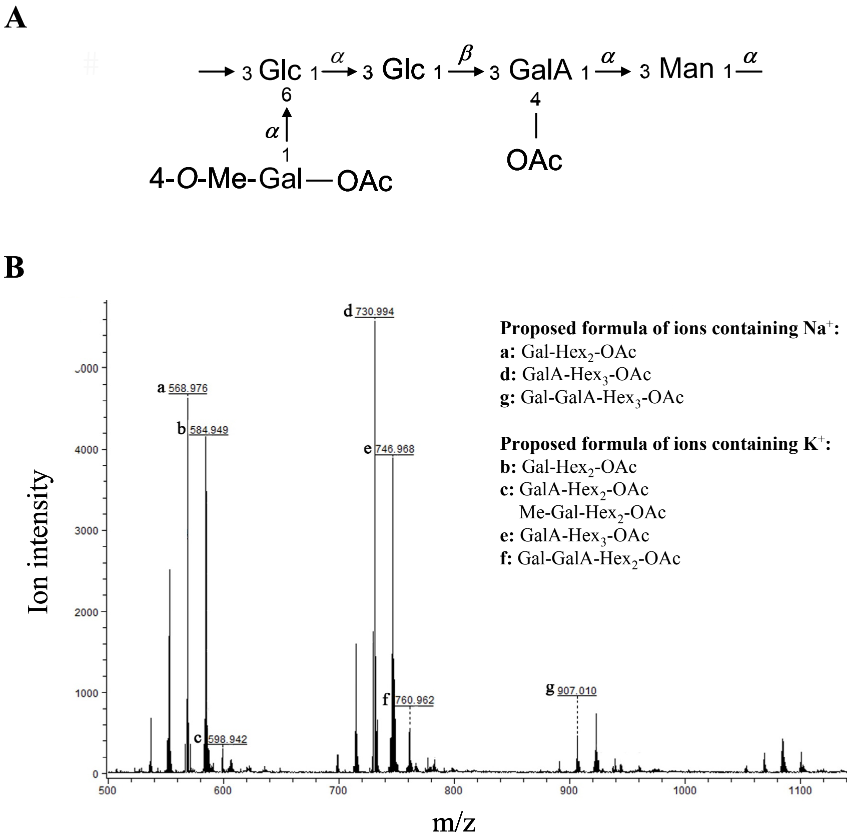

Figure S2. MALDI-TOF spectrum of oligosaccharides of *B. diazoefficiens* USDA110 derived from the ethanol-precipitated polysaccharide. The ethanol precipitate was purified by gel filtration, hydrolyzed with TFA and subjected to positive-ion MALDI-TOF mass spectrometry. (**A**) Repeating subunit of the EPS of strain USDA110 as reported by Mort & Bauer (1982). (**B**) Positive-ion MALDI-TOF mass spectrum of obtained oligosaccharides. Two major ion families were observed, corresponding to trisaccharides and tetrasaccharides of EPS. Most ions contained sodium and potassium adducts. Predicted and observed signals are listed in Table S2 along with their proposed formulas.

Mort A. J. & Bauer W. D. Application of two new methods for cleavage of polysaccharides into specific oligosaccharide fragments. Structure of the capsular and extracellular polysaccharides of *Rhizobium japonicum* that bind soybean lectin. Journal of Biological Chemistry 257, 1870-1875 (1982).

Table S1. Statistics of whole-genome short read re-sequencing results obtained from Tn5 mutant colonies.

Colony	No. of total reads	No. of mapped reads	Percentage of mapped reads (%)	Coverage position	Genome coverage (%)	Sequencing depth
BJ#25	39,732,425	39,630,023	99.74	8927192	98.0	557
BJ#47	47,978,094	47,830,501	99.69	8927360	98.0	671
BJ#57	42,575,145	42,430,556	99.66	8927154	98.0	589
WT	30,686,712	30,640,961	99.85	8926930	98.0	433

Short reads were mapped to the reference genome of *Bradyrhizobium diazoefficiens* USDA110 (NC_004463.1).
WT, wild-type USDA110.

Table S2. Predicted and observed mass spectrometry signals obtained from the MALDI-TOF spectrum shown in Figure S2.

Proposed formula[a]	$[M-Na]^{+1}$		$[M-K]^{+1}$	
	Predicted[b]	Observed[c]	Predicted	Observed
Me-Gal-Hex2-OAc	583.4746	NO[d]	599.5836	598.942
Gal-GalA-Hex3-OAc	907.7216	907.010	923.8306	NO
Gal-GalA-Hex2-OAc	745.5756	NO	761.6846	760.962
Gal-Hex2-OAc	569.4524	568.967	585.5614	584.949
GalA-Hex3-OAc	731.5984	730.994	747.7074	746.968
GalA-Hex2-OAc	583.4296	NO	599.5386	598.942

[a] Oligosaccharides corresponding to the published EPS structure containing hexose and galacturonic acid residues partially modified by methyl and *O*-acetyl groups.
[b] Values calculated from the average incremental masses.
[c] See Figure S2.
[d] NO, not observed.

Table S3. List of glycosyltransferase homologs of *blr2358* in the *Bradyrhizobium* genus.

See separate Excel file.

Table S4. Oligonucleotides used in this study.

ID	Name	Sequence, 5' to 3'
**Detection of pLG107-Tn5 plasmid insertion**		
OCX-1	2358HF	GCCTCTTCGCTATTACGCCA
OCX-2	2358HR	AGACGTCATACTCAACCCGC
**Expression of pBBR1MCS5-based *blr2358***		
OCX-3	pBBR1MCS5-blr2358_F	CGCTCTAGAACTAGTGGATCCTCAGTAGGCTTCCTGATGAA
OCX-4	pBBR1MCS5-blr2358_R	GTCGACGGTATCGATAAGCTTATGGCTGTAGCAACTTACGG

Table S5. Bacterial strains and plasmids used in this study.

Bacterial strain	Description	Reference or source
Bradyrhizobium diazoefficiens		
USDA110	Wild-type	Ref. 1
BJ#25	USDA110 Tn5 mutant colony	This study
USDA110-Ctrl	USDA110 carrying an empty pBBR1MCS5 plasmid	This study
BJ#25-pBBR1MCS5-*blr2358*	BJ#25 carrying the pBBR1MCS5-*blr2358* construct	This study
Escherichia coli		
DH5α	*fhuA2 Δ(argF-lacZ)U169 phoA glnV44 Φ80 Δ(lacZ)M15 gyrA96 recA1 relA1 endA1 thi-1 hsdR17*	Ref. 2
Plasmids		
pLG107-Tn5	Tn5-based transposon plasmid	Ref. 3
pBBR1MCS5	Broad-host-range expression plasmid	Ref. 4

1. Regensburger, B. & Hennecke, H. RNA polymerase from *Rhizobium japonicum*. Archives of Microbiology 135, 103-109 (1983).

2. Hanahan, D. *In* DNA Cloning: A Practical Approach (Glover, D.M., ed.), Vol. 1, p. 109, IRL Press, McLean, Virginia, USA (1985).

3. Pechter, K. B., Gallagher, L., Pyles, H., Manoil, C. S. & Harwood, C. S. Essential genome of the metabolically versatile alphaproteobacterium *Rhodopseudomonas palustris*. Journal of Bacteriology 198, 867–876 (2016).

4. Kovach, M. E., P. H. Elzer, D. S. Hill, G. T. Robertson, M. A. Farris, R. M. Roop II, and K. M. Peterson. Four new derivatives of the broad host range cloning vector pBBR1MCS, carrying different antibiotic-resistance cassettes. Gene 166, 175-176 (1995).
